# Evaluation of the Hyoid Bone Position Based on the Hyoid Triangle in Patients With Different Skeletal Patterns: A Lateral Cephalometry Cross‐Sectional Study

**DOI:** 10.1002/hsr2.72081

**Published:** 2026-04-05

**Authors:** Maryam Karandish, Mahvash Hasani, Sahar Dialameh

**Affiliations:** ^1^ Department of Orthodontics, School of Dentistry Shiraz University of Medical Sciences Shiraz Iran; ^2^ Department of Oral and Maxillofacial Radiology, School of Dentistry Shiraz University of Medical Sciences Shiraz Iran; ^3^ Department of Prosthodontics, School of Dentistry Qazvin University of Medical Sciences Qazvin Iran; ^4^ Student Research Committee Qazvin University of Medical Sciences Qazvin Iran

**Keywords:** cephalometric analysis, craniofacial morphology, growth pattern, hyoid bone position, orthodontics, skeletal patterns

## Abstract

**Background and Aims:**

We aimed to compare the hyoid bone triangle among individuals with Class I, II, and III skeletal patterns, considering different facial growth patterns.

**Methods:**

A total of 296 patients were included in the study. Skeletal patterns were classified using the A Point‐Nasion‐B Point (ANB) angle and Wit's appraisal, while the Bjork angle and Jarabak index were employed to categorize facial growth patterns (vertical, horizontal, and normal). Hyoid triangle measurements—based on two facial parameters (C3‐H and H‐RGn) and one cervical vertebral parameter (C3‐RGn)—were compared across skeletal classes and facial growth patterns.

**Results:**

Patients were classified into vertical (*n* = 143), horizontal (*n* = 80), and normal (*n* = 73) growth patterns. Significant differences in hyoid triangle measurements were observed across skeletal classes within each growth pattern. In the vertical group, mean C3–RGn was 71.90 ± 9.28 mm in Class I versus 65.40 ± 9.45 mm in Class II (*p* = 0.027). In the horizontal group, Class III exhibited significantly higher values than Class II for C3–H (34.19 ± 6.55 vs. 30.16 ± 4.18 mm, *p* = 0.050), H–RGn (93.13 ± 9.96 vs. 37.45 ± 6.02 mm, *p* = 0.007), and C3–RGn (76.56 ± 14.25 vs. 66.77 ± 9.11 mm, *p* = 0.008). In the normal group, Class III showed higher H–RGn (39.77 ± 7.72 vs. 33.57 ± 6.39 mm, *p* = 0.015) and C3–RGn (72.88 ± 11.27 vs. 63.89 ± 8.60 mm, *p* = 0.030) compared to Class II.

**Conclusion:**

Class II patients tend to have a more posterior hyoid bone position, except in normal growth patterns, where the hyoid position is relatively more anterior. Vertical growth patterns in Class II patients are associated with a lower hyoid bone position.

**Abbreviations**
ANB: A point‐nasion‐B Point
C3: third cervical vertebra
CBCT: cone beam computed tomography
FH: Frankfort horizontal
H: hyoidale
MP: mandibular plane
RGn: retrogenathion
SD: standard deviation
SN: sella‐nasion
SPSS: statistical package for the social sciences

## 1 Background

The hyoid bone, located in the anterior neck, is a unique structure suspended by muscles and ligaments without direct articulation to any other bones. It plays a critical role in swallowing, speech, and respiration [1]. The position of the hyoid bone is influenced by various factors such as head posture, suprahyoid and infrahyoid muscle activity, and craniofacial morphology [2, 3].

Changes in hyoid bone position have been associated with different skeletal patterns and malocclusion types [4, 5, 6]. For example, patients with Class II skeletal patterns often exhibit a more inferiorly and posteriorly positioned hyoid bone compared to Class I, which may influence upper airway dimensions and potentially lead to conditions like obstructive sleep apnea [7, 8]. Numerous studies have investigated how malocclusion, orthognathic surgery, and orthodontic treatment affect hyoid bone position [9, 10, 11].

Skeletal pattern, defined by the anteroposterior relationship between the maxilla and mandible, is a key factor influencing hyoid bone position. Previous studies have shown that the hyoid bone tends to be positioned more anteriorly in individuals with Class II skeletal malocclusion and more posteriorly in Class III ones [12, 13]. Wu et al. demonstrated significant variations in hyoid bone position based on facial growth patterns and dental ages, highlighting the relationship between facial morphology and hyoid bone position in Chinese adults [14].

Additionally, studies like Verma et al. [15] have shown anterior displacement of the hyoid bone following Twin‐block therapy, while Enacar et al. [10] reported posterior displacement after mandibular setback surgery in Class III patients. These findings emphasize the importance of understanding how hyoid bone position varies across skeletal patterns, as this may have implications for orthodontic treatment planning, airway management, and overall craniofacial development. To address the gap in knowledge, we adapted the hyoid triangle methodology for simultaneous application across the full spectrum of sagittal skeletal classifications (Class I, II, and III) and vertical facial growth patterns (vertical, horizontal, and normal). By integrating three key linear measurements (C3–H, H–RGn, and C3–RGn) into a unified triangular framework, we aimed to achieve a fully three‐dimensional representation of hyoid position relative to both mandibular and cervical landmarks. This comprehensive spatial mapping not only increases our biomechanical understanding of how craniofacial form and posture influence hyoid orientation, but also informs clinicians with a powerful tool for early identification of airway compromise, such as obstructive sleep apnea, through deviations in hyoid‐mandible‐cervical spatial relationships. Furthermore, by establishing normative “triangular envelopes” tailored to each combination of skeletal class and growth direction, our approach enables bespoke orthodontic and surgical planning, allowing practitioners to customize functional appliance design or surgical vectors in accordance with a patient's unique craniofacial and respiratory requirements. The novelty of our study is its ability to provide growth‐pattern–specific insights into hyoid positioning and its implications for clinical applications beyond orthodontics alone. While prior research has acknowledged associations between skeletal class and hyoid location, our triangulated framework allows for an interpretation of these relationships, showing that how the same skeletal class may present differently in terms of hyoid orientation depending on the vertical growth pattern. The aim of this study is to investigate the position of the hyoid bone in different skeletal patterns, particularly examining its variations in vertical, horizontal, and normal growth patterns, and its relationship with skeletal and dental features. This study seeks to provide new insights into how different malocclusion types and growth patterns affect hyoid bone positioning.

## 2 Methods

Following approval by the Shiraz Dental School Ethics Committee, this study recruited participants from patients referred to the Department of Orthodontics at Shiraz Dental School. The sample size, calculated from a pilot study, was designed to achieve a 95% confidence level with a maximum allowable error of 0.04. A total of 296 patients were ultimately included in this retrospective study. Patients were eligible for inclusion if they had been referred for orthodontic treatment and required lateral cephalometry for treatment planning. Exclusion criteria were applied to patients without a visible hyoid bone on lateral cephalometry, those with distorted lateral cephalometric images, and individuals with systemic or congenital conditions that might influence growth. Those with severe dental abnormalities that could influence skeletal measurements were excluded. Specifically, patients with complex malocclusions, including crossbite, deep bite, or open bite, were excluded from the study to ensure homogeneity in the sample and avoid potential confounding factors affecting hyoid bone position.

Lateral cephalometric radiographs were obtained for all participants and selected based on their quality and sharpness. All radiographs were taken using the same equipment and by the same technician to ensure consistency. Patients were positioned in natural head posture by having them stand and look directly into a mirror. A plumb line was suspended from the cassette to indicate the true vertical line. Measurements were conducted on 17.5 × 17.5 cm transparent films, using a 0.5 mm pencil, protractor, and adhesive tape for precision. All landmark identification and measurements were performed by a single examiner with extensive training in cephalometric tracing to ensure consistency. Formal intra‐examiner and inter‐examiner reliability testing was not performed.

### 2.1 Cephalometric Measurements

Patients were categorized based on their skeletal pattern and vertical growth pattern:

- Skeletal pattern classification (based on A Point‐Nasion‐B Point (ANB) angle and Wit's appraisal):

◦ Class I: ANB angle (2° to 5°) and Wit's measurement (−1, 0)
◦ Class II: ANB angle ( > 5°) and Wit's measurement ( > 0)
◦ Class III: ANB angle ( < 2° or negative) and Wit's measurement (<−1)

- Growth pattern classification (based on Bjork angle and Jarabak index):

◦ Vertical growth: Bjork angle ( > 401°) and Jarabak index ( < 62%)
◦ Horizontal growth: Bjork angle ( < 391°) and Jarabak index ( > 65%)
◦ Normal growth: Bjork angle (396° ± 5) and Jarabak index (62%–65%)

**Figure 1:**
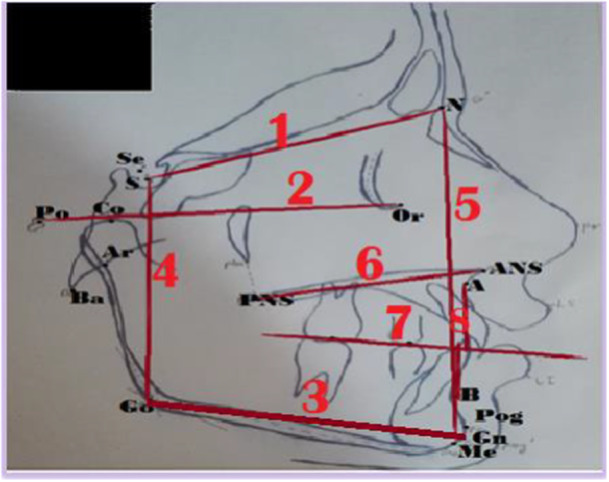
The cephalometric lines and plans.

**Figure 2:**
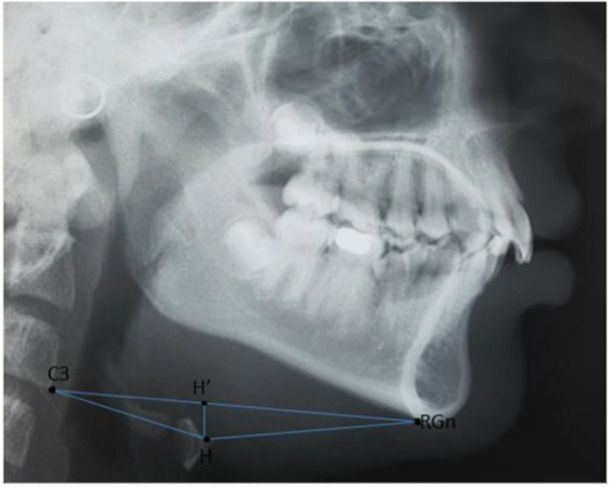
The cephalometric triangular measurements.

The definition of points, along with linear and angular measurements used in the present study, are shown in Table 1. Moreover, schematic illustrations of cephalometric lines and planes are provided in Figure 1. Figure 2 shows cephalometric triangular measurements.

**Table 1:** Cephalometric parameters and measurements.

Parameters	Definition
Points	
A (subspinale)	The deepest point along the midline of the premaxilla, positioned between the anterior nasal spine and prosthion.
B (supramentale)	The farthest posterior point within the hollow between the infradentale and pogonion.
S (sellaturcica)	The central point of the sella turcica is located in the hypophyseal fossa
N (nasion)	The midpoint of the frontonasal suture
ANS	The foremost midpoint of the anterior nasal spine of the maxilla
PNS	The posterior midpoint of the posterior nasal spine of the palatine bone
Pg (pogonion)	The anterior midpoint of the chin on the outline of the mandibular symphysis
Me (menton)	The inferior midpoint of the chin on the outline of the mandibular symphysis
Or (orbitale)	The inferior point of each infraorbital rim
Go (gonion)	The midpoint of the contour connecting the ramus and body of the mandible
Gn (genathion)	The most anterior and inferior point on the symphysis of the mandible
H (hyoidale)	The anterior superior point on the body of the hyoid
RGn (retrogenathion)	The posterior and inferior point on the mandibular symphysis
C3	The anteroinferior point on the third cervical vertebra
Lines and planes	
SN (sella‐nasion plane)	The line connecting S and N
FH (Frankfort horizontal plane)	The line connecting P and Or
MP (mandibular plane)	The line connecting Go and Gn
Occlusal plan	A line drawn from the molar to the premolar tooth.
H‐C3	Linear distance between H and C3
H‐RGn	Linear distance between H and RGn
C3‐RGn	Linear distance between C3 and RGn
Wit's appraisal	Liner measurement among lines dropped from A point and B point on the occlusal plane.
Jarabak index	The ratio between the posterior facial height (S‐Go) to the anterior facial height (N‐Me)
Angles	
Bjork angle	The angle between the SN line and the mandibular plane
SNA	An angle consisting of three points of S, N, and A
SNB	An angle consisting of three points of S, N, and B
ANB	The difference between SNA and SNB

### 2.2 Statistical Analysis

Data were analyzed using SPSS software (version 22) to determine the relationships between hyoid triangle measurements and various skeletal and growth patterns. Descriptive statistics, including means and standard deviations, were calculated for all variables.

To ensure the appropriate statistical tests were used, the normality of the data distribution was assessed for all variables. As the data were non‐normally distributed, nonparametric tests were selected for the group comparisons. A Kruskal–Wallis H test was performed to analyze differences in the linear measurements of the hyoid triangle (C3‐H, H‐RGn, and C3‐RGn) across the three skeletal classes (class I, II, and III) within each facial growth pattern group (vertical, horizontal, and normal). This test is the appropriate nonparametric alternative to a one‐way ANOVA for comparing more than two independent groups with non‐normal data.

Following significant results from the Kruskal–Wallis test (*p* < 0.05), post‐hoc pairwise comparisons with Bonferroni correction were conducted to identify which specific skeletal classes differed from one another. This was done to maintain the overall significance level and minimize the risk of a Type I error. The findings from these pairwise comparisons are detailed in the results section, specifying the significant differences between groups (e.g., Class I vs. Class II and Class II vs. Class III). Statistical significance was set at two‐sided *p* < 0.05 for all analyses.

## 3 Results

A total of 296 patients were studied and categorized into vertical, horizontal, and normal growth pattern groups based on cephalometric measurements. The mean age of the total sample was 27.3 ± 5.8 years (range: 18–45 years). The study included an equal distribution of genders (148 males and 148 females). A statistical comparison using the Kruskal–Wallis test showed no significant difference in mean age between the skeletal malocclusion groups (*p* < 0.05), indicating that age was not a confounding factor in the study results. In the vertical growth pattern group, 143 patients (47 Class I, 45 Class II, and 51 Class III) were included. In the horizontal growth pattern group, 80 patients (28 Class I, 24 Class II, and 28 Class III) were included. In the normal growth pattern group, 73 patients (25 Class I, 24 Class II, and 24 Class III) were included. This study evaluated the hyoid triangle measurements across different growth patterns. The sides of the hyoid triangle included linear measurements of C3‐H, H‐RGn, and C3‐RGn, which were compared between different skeletal patterns.

### 3.1 Vertical Growth Pattern

In the vertical growth pattern group (143 subjects), a statistically significant difference was found in the linear measurement of C3‐RGn between different skeletal patterns (*p *= 0.028). Pairwise comparison showed that the average value of C3‐RGn in patients with class I (71.90 ± 9.28) was significantly higher than in patients with class II (65.40 ± 9.45) skeletal pattern (*p *= 0.027). There were no significant differences between patients with different skeletal patterns in terms of mean C3‐H and H‐RGn measurements in the vertical growth pattern group (Table 2).

**Table 2:** Hyoid triangle measurements in the vertical growth pattern group.

Measurements	Mean ± SD			*p* value
	Class I (*n* = 47)	Class II (*n* = 45)	Class III (*n* = 51)	
C3‐H	32.477 ± 4.995	30.517 ± 4.417	32.325 ± 5.99	0.075
H‐RGn	37.863 ± 6.739	35.500 ± 6.223	38.034 ± 6.719	0.236
C3‐RGn	70.318 ± 9.395	65.410 ± 9.979	68.089 ± 8.737	0.028

### 3.2 Horizontal Growth Pattern

In the horizontal growth pattern group, there were statistically significant differences in C3‐H, H‐RGn, and C3‐RGn measurements across various skeletal patterns. Among these, class III and class II skeletal patterns exhibited the largest discrepancies in all three measurements. Specifically, the mean C3‐H value was significantly greater in class III skeletal pattern (34.19 ± 6.55) than in class II (30.16 ± 4.18) (*p *= 0.050). Furthermore, the most pronounced difference in H‐RGn was found between class III (93.13 ± 9.96) and class II (37.45 ± 6.02) (*p *= 0.007). For C3‐RGn, patients with a class III skeletal pattern demonstrated a significantly higher mean (76.56 ± 14.25) compared to those with a class II pattern (66.77 ± 9.11) (*p *= 0.008) (Table 3).

**Table 3:** Hyoid triangle measurements in the horizontal growth pattern group.

Measurements	Mean ± SD			*p* value
	Class I (*n* = 28)	Class II (*n* = 24)	Class III (*n* = 28)	
C3‐H	33.727 ± 6.001	30.161 ± 4.180	34.187 ± 6.554	0.028
H‐RGn	41.545 ± 7.365	37.45 ± 6.015	93.125 ± 9.955	0.025
C3‐RGn	74.757 ± 9.048	66.774 ± 9.106	76.562 ± 14.245	0.002

### 3.3 Normal Growth Pattern

In patients with a normal growth pattern, statistically significant differences were observed between skeletal patterns for H‐RGn and C3‐RGn measurements. However, no significant difference was found in mean C3‐H across skeletal patterns. Pairwise comparisons revealed that the mean H‐RGn value in class III patients (39.77 ± 7.72) was significantly higher than that in class II patients (33.57 ± 6.39) (*p* = 0.030). Additionally, the mean C3‐RGn measurement was significantly greater in patients with a class III skeletal pattern (72.88 ± 11.27) compared to those with a class II pattern (63.89 ± 8.60) (*p* = 0.030) (Table 4).

**Table 4:** Hyoid triangle measurements in normal growth pattern group.

Measurements	Mean ± SD			*p* value
	Class I (*n* = 25)	Class II (*n* = 24)	Class III (*n* = 24)	
C3‐H	31.947 ± 5.264	31.875 ± 3.326	34.153 ± 5.717	0.733
H‐RGn	39.684 ± 6.146	33.572 ± 6.385	39.769 ± 7.721	0.030
C3‐RGn	70.631 ± 8.719	63.892 ± 8.603	72.884 ± 11.268	0.030

### 3.4 Total Sample Comparison

When comparing skeletal classes (class I, II, and III), regardless of growth pattern, significant differences were observed in H‐C3, C3‐RGn, and H‐RGn measurements. The linear measurement of C3‐H was significantly different between all groups (*p *= 0.001). Pairwise comparisons showed the largest differences between Class I and II (*p *= 0.024) and between Class II and III (*p *= 0.020), with the lowest value corresponding to Class II. C3‐RGn measurement showed a significant relationship with skeletal patterns (*p *< 0.001). Pairwise comparisons indicated the largest difference between Class I and II (*p *< 0.001), and Class II and III (*p *= 0.030), with the lowest value corresponding to Class II. Similarly, the H‐RGn measurement also differed significantly between the three groups (*p *< 0.001). Pairwise comparisons showed the most significant differences between Class I and II (*p *< 0.001) and Class II and III (*p *= 0.021), with the highest value corresponding to Class I (Table 5).

**Table 5:** Hyoid triangle measurements in the total sample.

Measurements	Mean ± SD			*p* value
	Class I	Class II	Class III	
C3‐H	32.802 ± 5.401	30.730 ± 3.987	33.235 ± 6.017	0.001
H‐RGn	39.489 ± 6.978	35.556 ± 6.309	35.511 ± 8.094	< 0.001
C3‐RGn	71.906 ± 9.287	65.408 ± 9.405	70.129 ± 11.442	< 0.001

## 4 Discussion

This study employed lateral cephalometry to assess the linear measurements of the hyoid triangle across distinct facial growth patterns: vertical, horizontal, and normal. Participants were categorized based on anteroposterior dentofacial discrepancies, using the ANB and Wits angles, which are widely recognized parameters in related research [16]. Due to limited software accessibility and a lack of expertise among students, lateral cephalometric analyses were conducted using manual tracing. This approach aligns with prior studies by Jena et al. [17] and Sayinsu et al. [18], whereas digital methods, such as those implemented by Polat‐Ozsoy et al. [19], were not applied.

While some researchers have utilized cone‐beam computed tomography (CBCT) [20], lateral cephalometry was selected for this study based on its accessibility and the ease of identifying anatomical landmarks in 2‐D imaging. Our results showed an increased C3‐H in Class III patients relative to those in Class I and II, which might be due to the jaw posture in Class III patients. This finding aligns with Abu Allhaaij et al. [21], who reported a shorter H‐C3 distance in Class II patients compared to Classes I and III. Similarly, research by Saeeda et al. indicated that Class II patients exhibited a more posteriorly positioned hyoid bone [22]. Conversely, Soheilifar et al. found no significant differences in the horizontal or vertical positioning of the hyoid bone between Class I and II groups, suggesting that compensatory adjustments within surrounding soft tissues may play a role [23]. These findings align closely with those of the current study. Specifically, regardless of growth pattern, the H‐RGn distance was consistently lowest in Class II, indicating a link between mandibular retrusion and the forward positioning of the hyoid bone. Furthermore, H‐C3 measurements were also lower in Class II than in other skeletal classes, suggesting a more posterior position of the hyoid bone. These observations are supported by recent CBCT studies, which also note a posterior hyoid position in Class II individuals [24]. These observations imply that such shifts can be attributed to a reduction in the C3‐RGn measurement within Class II, which results from mandibular retrusion, thereby leading to a corresponding posterior displacement of the hyoid bone. Our study's results are consistent with those of Amayeri et al. [25], who also reported a greater H‐C3 distance in Class III patients. Similarly, Mortazavi et al. [4] observed the smallest H‐C3 distance in Class II patients, corroborating our findings. Additionally, our results for H‐RGn revealed significant differences, with Class II patients showing shorter distances compared to Class I. This supports the findings of Ferraz et al. [26] but contrasts with those reported by the study by Amayeri et al. [25].

This study also explored variations by growth pattern. Among patients with vertical growth patterns, Class II exhibited the lowest C3‐RGn measurement, while C3‐H and H‐RGn did not differ significantly from other classes, suggesting a lower hyoid bone position in vertical growth patients. In those with a horizontal growth pattern, all three measurements—H‐C3, H‐RGn, and C3‐RGn—were lower in Class II than in Class III, indicating that mandibular retrusion in this class contributed to the proportional posterior positioning of the hyoid bone. In patients with a normal growth pattern, despite lower C3‐RGn and H‐RGn measurements in Class II, no significant difference was observed in H‐C3 between Classes II and III. This suggests that mandibular retrusion does not proportionally affect the hyoid bone's posterior position, resulting in a more anterior hyoid location in these individuals.

Adil et al. observed a lower hyoid position in hypodivergent patients compared to other growth patterns, which agrees with our findings, as a lower hyoid bone position was identified only in Class II patients with a vertical growth pattern [27]. Additionally, a study using cone beam computed tomography by Chen et al. found that skeletal Class II patients exhibited a posterior‐inferior hyoid bone position compared to other skeletal classes [28]. Our results for Class II patients are also consistent with the findings of Chen et al. [28]. However, our findings indicated that in patients with a normal growth pattern, the hyoid bone is positioned more anteriorly in class II. Slight differences in results might be due to the fact that in the previous study, growth patterns of patients were not considered. Furthermore, the study by Singh et al. [24], which also used CBCT, corroborated these findings by reporting that the hyoid bone is posteriorly positioned in Class II skeletal patterns and that oropharyngeal volume is least in these individuals [24]. The growing trend towards three‐dimensional imaging for craniofacial analysis, as seen in studies like the one by Çelik et al. [29], highlights the need for a comprehensive approach to understanding these complex relationships [29].

This altered hyoid posture has direct ramifications for airway patency. A posterior‐inferior hyoid reduces oropharyngeal volume and may elevate the risk of obstructive sleep apnea by narrowing the hypopharyngeal airway. Recent studies using advanced imaging techniques have also linked increased tongue volume and hyoid bone volume, which can be a key consideration in dentomaxillary development and surgical planning [30]. Furthermore, inferior hyoid positioning can influence tongue posture and resting muscle tone, potentially exacerbating airway collapse during sleep. Clinicians should consider these dynamics when planning orthodontic or surgical interventions. For example, functional appliances that advance the mandible (Twin‐block, Herbst) may simultaneously reposition the hyoid forward, enhancing airway dimensions. Customizing appliance design to each patient's growth pattern, such as increasing vertical opening in hyperdivergent Class II cases, could optimize both skeletal correction and respiratory function. These insights also inform surgical planning by quantifying expected changes in the hyoid triangle that allow prediction of postoperative airway effects following mandibular advancement or setback. An interdisciplinary approach through integrating cephalometric hyoid analysis with polysomnography can guide more targeted therapies for patients presenting with both malocclusion and sleep‐disordered breathing.

### 4.1 Limitations and Future Directions

The study sample, sourced from a single dental school, may limit the generalizability of the findings. It is also important to note that this cross‐sectional study provides a snapshot in time, with further longitudinal studies needed to track changes over time. Linear measurements may also fail to fully capture the hyoid bone's three‐dimensional relationships. Another limitation of this study is the absence of formal intra‐ and inter‐examiner reliability testing. Although all cephalometric tracings and measurements were performed by a single trained examiner to minimize variability, the lack of repeatability assessment may introduce potential measurement bias. Future studies should incorporate systematic reliability testing to ensure reproducibility and enhance methodological rigor. Next studies should also consider advanced imaging techniques, such as CBCT, for a more comprehensive evaluation of hyoid bone positioning and its relevance for orthodontic diagnosis and treatment planning. Recognizing these variations allows clinicians to tailor treatment strategies to each patient's unique craniofacial anatomy, potentially optimizing outcomes. Further studies may build on these findings to refine orthodontic methods and better address patient‐specific needs.

## 5 Conclusions

The current study revealed distinct positional differences among skeletal classes, which could enhance insights for orthodontic treatment planning. Generally, Class II patients show a more posterior hyoid bone position, except those with a normal growth pattern. Only Class II patients with a vertical growth pattern demonstrated a lower hyoid position.

## Author Contributions

**Maryam Karandish:** writing – review and editing, methodology, conceptualization, investigation, resources, project administration, supervision. **Mahvash Hasani:** writing – review and editing, methodology, conceptualization, investigation, resources, project administration. **Sahar Dialameh:** data curation, investigation, methodology, writing – original draft.

## Funding

The authors have nothing to report.

## Ethics Statement

This study was approved by the Shiraz Dental School Ethics Committee (approval reference number: IR.SUMS.REC.1397.505). All participants provided informed consent prior to inclusion in the study. The proposal of the current study was approved by the ethical committee of the vice chancellor for research, Shiraz University of Medical Sciences (ethics code: 1397.505) and the researchers confirm the adherence to ethical guidelines.

## Consent

Informed consent was obtained from all patients who participated in this study.

## Conflicts of Interest

The authors declare no conflicts of interest.

## Transparency Statement

The corresponding author, Sahar Dialameh, affirms that this manuscript is an honest, accurate, and transparent account of the study being reported; that no important aspects of the study have been omitted; and that any discrepancies from the study as planned (and, if relevant, registered) have been explained.

## Data Availability

The data sets generated and analyzed during the current study are available from the corresponding author upon reasonable request via email.
